# Crosstalk between cytokinin and ethylene signaling pathways regulates leaf abscission in cotton in response to chemical defoliants

**DOI:** 10.1093/jxb/erz036

**Published:** 2019-02-04

**Authors:** Jiao Xu, Lin Chen, Heng Sun, Nusireti Wusiman, Weinan Sun, Baoqi Li, Yu Gao, Jie Kong, Dawei Zhang, Xianlong Zhang, Haijiang Xu, Xiyan Yang

**Affiliations:** 1 National Key Laboratory of Crop Genetic Improvement, National Center of Plant Gene Research (Wuhan), College of Plant Science and Technology, Huazhong Agricultural University, Wuhan, PR China; 2 Institute of Economic Crops, Xinjiang Academy of Agricultural Sciences, Urumqi, Xinjiang, PR China

**Keywords:** Cotton, cytokinin signaling, defoliant, ethylene signaling, GhCKX3, leaf abscission

## Abstract

Abscission is a process that allows plants to shed tissues or organs via cell separation, and occurs throughout the life cycle. Removal of leaves through the use of chemical defoliants is very important for mechanical harvesting of cotton (*Gossypium hirsutum*). However, our knowledge of the molecular mechanisms of the defoliation response involved is limited. In this study, RNA-seq was conducted in order to profile the differentially expressed genes (DEGs) between cultivars X50 (sensitive to chemical defoliants) and X33 (relatively insensitive) at different time points after treatment with thidiazuron and ethephon (TE). A total of 2434 DEGs were identified between the two cultivars across the different time-points. Functional categories according to GO and KEGG analyses revealed that plant hormone signal transduction and zeatin biosynthesis were involved in the response to TE. Cytokinin oxidase/dehydrogenase (CKX) genes and ethylene-related genes were up-regulated following TE treatment, and were associated with increased level of ethylene, especially in cultivar X50. Down-regulation of *GhCKX3* resulted in delayed defoliation and a reduced ethylene response. The results show that crosstalk between cytokinin and ethylene regulates cotton defoliation, and provide new insights into the molecular mechanisms underlying the mode of action of defoliants in cotton.

## Teaser

Enhanced cytokinin signaling results in delayed defoliation and a reduced response to ethylene in cotton treated with thidiazuron and ethephon, with crosstalk between cytokinin and ethylene signaling playing an important regulatory role.

## Introduction

Abscission is a process whereby plant organs such as flowers, leaves, and fruit separate from the plant, for example when plants are subject to environmental stress or undergo senescence as part of their life cycle. The abscission pathway has been proposed to comprise of four major steps, namely determination of the abscission zone, competence to respond to abscission signals, activation of abscission for organ shedding, and differentiation of a protective layer post-abscission (Patterson, 2001).

Studies have shown that several signaling pathways are involved in the control of organ abscission. The leucine-rich repeat receptor kinase (LRR-RK) gene *HAESA* (*HAE*) is expressed in the abscission zone and the reduction of function of HAE results in delayed abscission of floral organs in Arabidopsis (Jinn *et al*., 2000). The binding of the peptide hormone INFLORESCENCE DEFICIENT IN ABSCISSION (IDA) to *HAE* activates a MAPK cascade to regulate cell separation in the abscission zone (Cai and Lashbrook, 2008; Santiago *et al*., 2016). The MADS-box domain proteins AGL15 and AGL18 participate in Arabidopsis floral shedding: AGL15 is a regulator of *HAE*, binding to its promoter, and is differentially phosphorylated by a MAPK cascade (Adamczyk *et al*., 2007; Patharkar and Walker, 2015). Somatic embryogenesis receptor-like kinases (SERKs), including SERK1, SERK2, SERK3, and SERK4, are co-receptors of HAE in the perception of IDA and act as negative regulators of floral organ abscission (Lewis *et al*., 2010; Meng *et al*., 2016). In addition, a number of transcription factors have been identified that regulate the development of the abscission zone. *JOINTLESS* and *MACROCALYX* (*MC*) encode MADS-box transcription factors. The mutation *jointless* suppresses the development of the abscission zone in tomato, and *JOINTLESS* and *MC* interact in the regulation of floral and fruit organ abscission zones development (Mao *et al*., 2000; Nakano *et al*., 2012). The expression of Arabidopsis *ZINC FINGER PROTEIN2* (*AtZFP2*) is elevated in the sepal abscission zone and it participates in organ shedding (Cai and Lashbrook, 2008). In cassava, 10 *R2R3 MYB* transcription factors have been found to be expressed in abscission zones during leaf abscission induced by ethylene and water deficit, and they are involved in the responses to stress and ethylene and auxin stimuli (Liao *et al*., 2016*b*).

Phytohormones are considered to play important roles in regulating leaf abscission (Gomez-Cadenas *et al*., 1996; Patterson and Bleecker, 2004). One important regulator is ethylene (Jackson and Osborne, 1970). Dynamic changes in endogenous ethylene have been detected during leaf abscission in cotton (Suttle, 1985). Ethylene-induced abscission is associated with important processes including cell wall modification, hormone signal transduction, lipid transport, and lignin biosynthesis, which lead to increased expression of cellulase and ethylene biosynthesis enzymes in the abscission zones (Agusti *et al*., 2008; Mishra *et al*., 2008*b*). Phospholipid signaling also plays an important role in regulating leaf and fruit abscission; for example, in *Citrus sinensis* the phospholipid signaling gene phospholipase D (*CsPLDγ1*) is induced by the ethylene stimulant ethephon (Malladi and Burns, 2008). Furthermore, lignin biosynthesis and the honeycomb structure of lignin supports the controlled degradation of the cell wall and enables precise cell separation in the abscission zone. Accumulation of H_2_O_2_ and superoxide is required for lignin formation, and Arabidopsis *rbohD/F* mutants lack lignin formation and exhibit delayed abscission (Lee *et al*., 2018). Reactive oxygen species (ROS) are therefore necessary for proper organ separation. Overexpression of the ROS-scavenging proteins SOD and CAT1 also delays cell separation (Liao *et al*., 2016*a*).

Auxin signaling is also proposed to be involved in the regulation of organ abscission. *Auxin response factor 2* (*ARF2*), *Auxin resistant 1* (*AUX1*), and *Like-Auxin3* (*LAX3*) are specifically expressed in the abscission zones of Arabidopsis. The *arf2* mutation delays the abscission of floral organ (Ellis *et al*., 2005; Basu *et al*., 2013). Auxin transporters regulate leaf shedding via inhibition of polar auxin transport in abscission zones (Suttle, 1988; Jin *et al*., 2015). The expression of all genes leading to to pedicel abscission, including those that are ethylene-induced, can be inhibited by application of IAA. Taken together, the available data indicate multiple layers of complexity in auxin, cytokinin, and ethylene crosstalk. Auxin–ethylene crosstalk has been defined as one of the most important regulatory pathways in the control of abscission at the molecular level (Meir *et al*., 2010; Liu *et al*., 2017).

Cotton (*Gossypium hirsutum*) is an important commercial crop harvested for its excellent natural fibers. As a result of changes in agricultural practice and the development of large-scale farming, machine-picking methods now dominate in the production of cotton. Removal of leaves before harvesting of the cotton bolls is an important agronomic practice, especially for mechanical harvesting, and hence the selection of appropriate chemical defoliants and suitable crop varieties for machine-picking are critical factors for cotton production. Thidiazuron (a synthetic cytokinin-like molecule) is a defoliant that is widely used in agriculture to facilitate mechanical harvesting for many crops, especially cotton. Some cotton varieties have been selected specifically as machine-picking cultivars for their fast response to defoliants and have been planted extensively across large areas. For example, Xinluzao 50 (X50) is one such cultivar and it has an ideal plant type, with strong growth potential, strong boll-setting, good fiber quality, and high resistance to wilt disease, and it is widely planted in Xinjiang province, the biggest cotton-cultivation area in China (Xu *et al*., 2011). However, despite the development of varieties with differing sensitivity to defoliants, the mechanisms underlying the response are not well understood.

In this study, two cotton cultivars, X50 and Xinluzao 33 (X33), were selected for their differing sensitivities to a defoliant treatment, namely a mix of thidiazuron and ethephon (together termed TE) that is widely used in the field for mechanical harvesting of cotton (Xu *et al*., 2011). X50 is more sensitive to TE treatment than X33. The transcriptomes of the abscission zones of the two cultivars at different time points following TE treatment and water treatment (W) were examined. GO and KEGG pathway analyses was used to characterize the signaling and gene expression responses, and a functional analysis of key genes then revealed new mechanistic insights into abscission signaling.

## Materials and methods

### Plant material and growth conditions

The cotton (*Gossypium hirsutum* L.) material used included Xinluzao 50 (X50), Xinluzao 33 (X33), and two transgenic lines RNAi lines with suppressed expression of *GhCKX3* (CR-3, CR-13) together with their wild-type control (WT, cv. Jimian 14), as described previously (Zhao *et al*., 2015). X50 is a high-quality and high-yielding variety bred by Xinjiang Academy of Agricultural Sciences, and is sensitive to defoliants and hence suitable for mechanized cultivation (Xu *et al*., 2011). X33 is relatively insensitive to defoliants. X50 and X33 were cultivated under normal agricultural conditions in the field in Changji (Xinjiang Province, China) and in a greenhouse at Huazhong Agricultural University (Wuhan, Hubei Province, China). *GhCKX3*-RNAi transgenic lines and a control WT line were planted in the field and in a greenhouse of glass and brick construction of ~60 m^2^ size at Huazhong Agricultural University. The greenhouse was subject to controlled conditions with a 12/12 h photoperiod and 30±2/25±2 °C day/night temperature. The leaf abscission zones were sampled carefully from the second and third youngest leaves at the boll-opening stage. All samples were either fixed in 50% formalin–acetic acid–alcohol (FAA) (Yang *et al*., 2012) or frozen immediately in liquid nitrogen.

### Defoliant treatment

The X50 and X33 cultivars were sown in the field and cultivated until the boll-opening stage. Then, either a commercial defoliant (a mixture of 400 mg l^−1^ thidiazuron and 2 ml l^−1^ 40% ethephon, together termed TE), 400 mg l^−1^ thidiazuron alone (T), or 2 ml l^−1^ 40% ethephon alone (E) were applied evenly to the leaves of the plants. The control consisted of an application of water (W). The TE treatment is a conventional defoliant application used agriculturally in Xinjiang Province and it is a good defoliant for the X50 cultivar (Xu *et al*., 2011). Leaf abscission zones were sampled at 1, 3, and 5 d after the treatments. Defoliation rates were calculated as the fold-change reduction in the number of leaves recorded at 1 week after treatment. Each experiment was performed with three biological replicates. Statistical analyses were conducted using the SPSS software.

The *GhCKX3*-RNAi transgenic lines (CR-3, CR-13) and the WT control line were planted in the field and in the greenhouse at Huazhong Agricultural University. The plants were treated with W and TE at the boll-opening stage and abscission zones were sampled at 1, 3, and 5 d. The samples were immediately frozen in liquid nitrogen until further analysis.

### Histological analysis

Histological observations were carried out to determine the sensitivities of X50 and X33 to the defoliant treatments and to observe the structure of abscission zones. The samples were fixed in 70% FAA and cleared with tertiary butanol. They were then embedded in paraffin and cut into 8-μm thick sections and stained with 1% Toluidine Blue as described previously (Xu *et al*., 2018). Images were obtained using a light microscope (Zeiss, Oberkochen, Germany).

### RNA extraction and sequencing

Twenty-four samples were used for RNA sequencing (RNA-seq) using two cultivars, X50 and X33; two treatments, defoliant (TE) and water control (W); three development stages, 1, 3, and 5 d; and two biological replicates per treatment. Total RNA was extracted from the abscission zones using a Spectrum Plant Total RNA Kit (Sigma).

Approximately 5 μg of total RNA samples were sent to Novogene for RNA sequencing. RNA quality was assessed before library construction, using the Agilent Bioanalyzer 2100 system. mRNA was purified from the total RNA. Libraries were sequenced on an Illumina HiSeq platform and 150-bp paired-end reads were generated. Clean reads were obtained by removing low-quality reads, reads containing adapters, and reads containing poly-N from raw reads. All the clean reads were aligned to the cotton genome (*G. hirsutum* TM-1 (AD)_1_) (Zhang *et al*., 2015). The mapped sequenced reads of the identified genes were used for gene expression analysis. Gene expression levels were calculated based on the FPKM (expected number of fragments per kilobase of transcript sequence per millions base pairs sequenced) (Trapnell *et al*., 2010).

### Analysis of DEGs

Using the statistical test padj <0.0001, a gene expressional value FPKM (max)>50 and absolute value |log_2_ ratio|>2 were considered to indicate significantly differentially expressed genes (DEGs). The DEGs were annotated with the *Arabidopsis thaliana* protein database and the NCBI non-redundant protein database. All DEGs were subjected to Gene Ontology (GO) enrichment analysis by Blast2GO with FDR<0.001 and were tested for statistical enrichment in Kyoto Encyclopedia of Genes and Genomes (KEGG) pathways using the KOBAS 2.0 software with *P*<0.001. Significant pathways were selected. Clustered analysis of DEG expression patterns was conducted using the Genesis K-means method (Sturn *et al*., 2002).

### qRT-PCR analysis

Candidate transcriptomic genes from the RNA-seq were selected and validated by qRT-PCR. Primers were designed using Primer Premier 5.0 and synthesized commercially (Genscript Bioscience, Nanjing, China) with the product size ranging from 100–250 bp. The first-strand cDNA was synthesized from 3 μg of total RNA using SuperScript III reverse transcriptase (Invitrogen). qRT-PCR was performed in 20-μl reactions using an Applied Biosystem 7500 real-time PCR system. Gene expression was calculated and normalized using *GhUBQ7* (DQ116441) as the internal control (Tu *et al*., 2007). All the reactions were performed with three biological replicates. The primers used for qRT-PCR are listed in Supplementary Table S7 at *JXB* online.

### ACC treatment and quantification of ethylene content

To further investigate the effects of ethylene on the X50 and X33 cultivars, seedlings were treated with different concentrations of 1-aminocyclopropane-1-carboxylic acid (ACC, Sigma). Sterilized seeds of cultivars were germinated on half-strength Murashige and Skoog medium (half macro salts plus 15 g l^–1^ glucose, pH 6.0) for 1 d. The germinated seedlings were then transferred into half-strength medium supplemented with either 0, 10, or 20 μM ACC and cultured at 28 °C in the dark for 5 d. The ACC was dissolved in sterilized water. The lengths of hypocotyls were measured and samples imaged using a Nikon D40 camera.

For measurement of ethylene evolution, abscission zone explants were excised from the two cultivars. These samples consisted of ~1 cm segments of the main stem and were collected in 1, 3, or 5 d after the W, TE, T, and E treatments (Suttle, 1985). Samples were sealed in 12-ml vials with air-tight corks at room temperature for approximately 24 h, then 1 ml of gas from the vial was sampled and injected into a gas chromatograph (7890A-5975C, Agilent), as described previously (Wang *et al*., 2018).

### Extraction and quantification of endogenous cytokinin

The concentration of endogenous cytokinins was determined in the abscission zones in X50, X33, the *GhCKX3*-RNAi transgenic lines (CR-3, CR-13), and the WT control line. The experiments were performed as described previously (Zeng *et al*., 2012) with modifications. Samples were extracted in 80% cold methanol overnight at 4 °C, which contained [^2^H_5_] trans-Zeatin (Olchemim, Olomouc, Czech Republic) and [^2^H_6_] N^6^-isopentenyladenine (Olchemim) as internal standards. After centrifugation at about 13 200 *g* for 15 min at 4 °C), the supernatant was passed through Oasis HLB columns (Waters). The filtered liquid was then evaporated using N_2_, dissolved in 2 ml 1% formic acid, and passed through Oasis MCX columns (Waters). The columns were first washed with 2 ml methanol, and then washed with 2 ml 0.35 M NH_4_OH. Finally, the columns were washed with 0.35 M NH_4_OH in 60% methanol and the elution was collected and evaporated using N_2_. The residue was dissolved in 100 μl 50% acetonitrile and stored at –80 °C until measurement. Cytokinins were quantified using a HPLC-MS/MS system with trans-Zeatin (Sigma) as the external standard.

### H_2_O_2_ determination

About 0.2 g of abscission zone samples were ground in liquid nitrogen, extracted in 1 ml 80% acetone, and shaken for 20 min in the dark, followed by centrifugation at 13 200 *g* at 4 °C for 15 min. The supernatant was used for measurement of H_2_O_2_, using a H_2_O_2_ Quantification Assay Kit (Sangon Biotech, Shanghai, China). After reaction with Fe^2+^, the absorbance at 595 nm was measured with a Multimode Plate Reader (PerkinElmer). All the reactions were performed with three biological replicates.

### Statistical analyses

All statistical analyses were conducted with at least three biological replicates, using Student’s *t*-test. Multiple comparisons were performed using the SPSS software.

## Results

### Dynamics of leaf abscission in response to defoliants

**Fig. 1.**
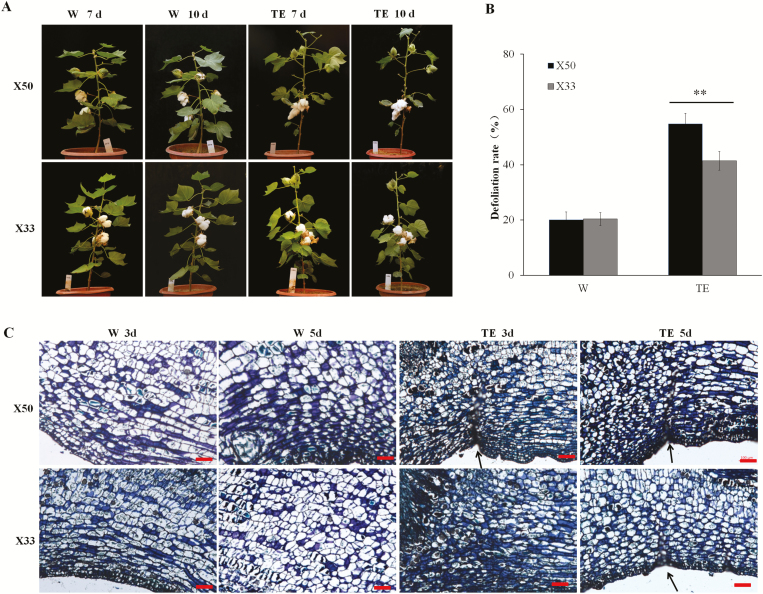
Comparison of defoliant sensitivity in X50 and X33 cotton cultivars. (A) Representative images of plants grown in the greenhouse taken at 7 d and 10 d following treatment with either thidiazuron and ethephon (TE) or water (W, control). (B) Defoliation rate at 7 d after treatment. The data are means (±SE), *n*=3. Significant differences were determined using Student’s *t*-test: **P<0.01. (C) Longitudinal sections of abscission zone regions under bright field conditions for plants grown in in the field at 3 d and 5 d following treatment. Formation of an abscission zone is indicated by the arrows. The sections are stained with toluidine blue. Scale bars are 100 μm.

To examine the mechanisms of leaf abscission under defoliant treatments in cotton, two cultivars with differing sensitivities to a commercial defoliant (a mixture of thidiazuron and ethephon, TE) were used, namely X50 (sensitive) and X33 (tolerant). Changes in morphology and histology were determined at the ball-opening stage both in the field and in a controlled greenhouse environment over 10 d following TE treatment. In addition, individual treatments of thidiazuron (T) and ethephon (E) treatments were also applied, with a water application (W) used as a control. Leaves were still green at 10 d following spraying with water, and no differences were observed between the two cultivars (Fig. 1A). Leaf abscission was accelerated as a result of the TE treatment. Some green leaves were dropped on X50 at 7 d post treatment, with a defoliation rate of ~54.8%. Some leaves also began to abscise on X33 at 7 d, with a defoliation rate of ~37% (Fig. 1B).

Similar phenotypes were seen for X50 and X33 following the TE and W (control) treatments. However, the defoliation rates across all four treatments were different. The defoliation rate following TE treatment in the field experiment was significantly increased in X50 compared with X33 (Supplementary Table S1). The defoliation rates of the TE and T treatments showed highly significant differences compared to the control (W), while the E treatment induced no difference at 7 d. The TE treatment, with a synergistic effect of thidiazuron and ethephon, caused the greatest defoliation of all the treatments. Paraffin-sectioning revealed that the formation of the abscission zone following TE treatment occurred at 3 d in X50, but it was not observed until 5 d in X33 (Fig. 1C). These divergent dynamics between X33 and X50 were then utilized to further analyse the mechanisms of leaf abscission in response to defoliant treatments by means of transcriptome analysis.

### Changes in the global transcriptome in the abscission zone in response to defoliants

To determine the global transcriptome profile of cotton in response to defoliants, RNA-seq (totalling 24 libraries) was performed for cultivars X50 and X33 at 1, 3, and 5 d following the TE and W treatments. Approximately 50 million paired-end reads and at least 40 million clean reads for each sample were obtained (Supplementary Table S2), and the clean reads were mapped to the *G. hirsutum* reference genome. We found that 85–89% of reads were totally mapped in those samples, of which 78–82% were uniquely mapping reads. The totally mapped reads were used to calculate gene expression levels based on FPKM for further analysis of DEGs (Trapnell *et al*., 2010).

**Fig. 2.**
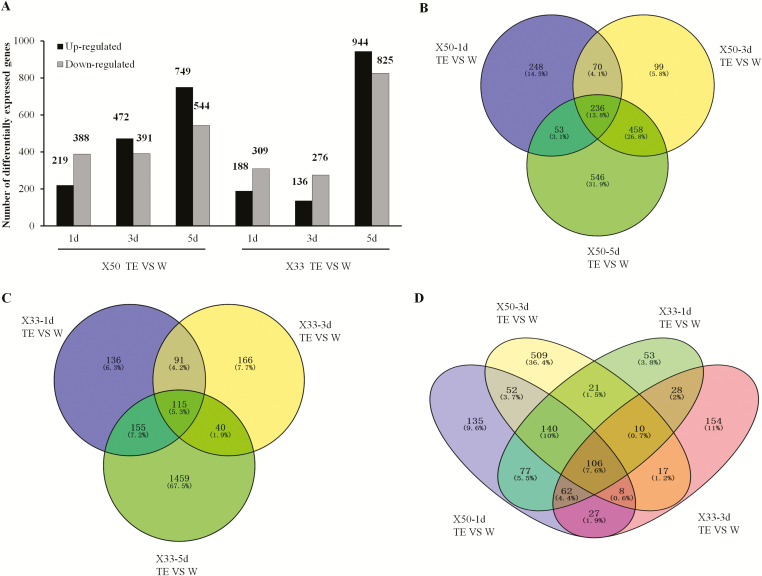
Differentially expressed genes (DEGs) in cotton cultivars X50 and X33 following treatment with either thidiazuron and ethephon (TE) or water (W, control) at different time-points. (A) The number of DEGs up- or down-regulated at 1, 3, and 5 d following treatment. (B, C) Venn diagrams for the number of DEGs at different points in (B) X50 and (C) X33. (D) Venn diagram showing a cross-comparison between cultivars for the number of DEGs at 1 d and 3 d following TE treatment.

More than 70 000 genes were expressed in the cotton abscission zone following the defoliant treatment, of which 2434 were DEGs were detected (1710 genes in X50, 2162 genes in X33) (Supplementary Table S3). The number of DEGs in X50 gradually increased following TE treatment, and was more than that in X33 in the earlier time-points (Fig. 2A). Compared to the control (W) treatment, there were more down-regulated genes at 1 d following TE treatment in cultivar X50, and more up-regulated genes at 3 d and 5 d. By contrast, in the relatively insensitive cultivar X33, more genes were down-regulated at 1 d and 3 d following treatment, and both the number of up- and down-regulated DEGs increased sharply at 5 d.

For X50, a total of 236 DEGs were expressed at all three time-points, 248 were expressed only at 1 d, 99 only at 3 d, and 546 only at 5 d (Fig. 2B). For X33, only 115 DEGs were expressed at all three time-points, whilst 136 were only expressed at 1 d, and 166 only at 3 d (Fig. 2C); however, there were 1459 genes expressed only at 5 d. We also compared the DEGs in X50 and X33 at 1 d and 3 d following TE treatment (Fig. 2D). More genes were either up- or down-regulated in X50 following treatment at both time-points. We also found that a great number of DEGs were distinctive between X50 and X33 (135 in X50-1d, 509 in X50-3d, 53 in X33-1d, 154 in X33-3d). The results suggested that the genes responding to the defoliant very distinct between the two cultivars in the early days following TE treatment.

The expression patterns of the total number of 2434 DEGs that we selected could be divided into three groups according to the Genesis software based on K-means clustering (Supplementary Fig. S1). Comparing the TE treatment with the W control, Type I DEGs (1122 genes) were down-regulated in both the X50 and X33 cultivars. These DEGs were divided into three clusters: cluster 1 (359 genes) was down-regulated by similar amounts at all three time-points; cluster 2 (232 genes) was down-regulated to a greater degree, especially in the X33 at 5 d; and cluster 3 (531 genes) showed a more progress down-regulation from 1 d to 5 d following treatment in both cultivars. Type II DEGs (716 genes) were moderately up-regulated in both cultivars following TE treatment and could also be divided into three further clusters: cluster 4 (130 genes) was up-regulated at 1 d but differential expression had virtually ceased at 5 d; cluster 5 (312 genes) showed no initial differential expression but was up-regulated gradually following TE treatment, with a continuous increase in X50 and a sharp increase in X33 between 3 d and 5 d; cluster 6 (274 genes) showed up-regulation at 1 d following TE treatment and this had increased a little by 5 d. Type III DEGs (596 genes) showed much more pronounced up-regulation following TE treatment in both X50 and X33 cultivars, and were again divided into three further clusters: cluster 7 (191 genes) was up-regulated at 1 d in both cultivars, with a steady increase to 5 d in X50, whilst X33 showed a dip at 3 d but then a sharp increase at 5 d; cluster 8 (90 genes) was greatly up-regulated at 1 d and showed a slight decline to 5 d in both cultivars; and cluster 9 (315 genes) was similar to cluster 5 but there was a greater increase in up-regulation by 5 d. Overall, the analysis showed that the expression of all DEGs in the response to the TE defoliant in cultivar X33 was delayed compared with X50.

**Fig. 3.**
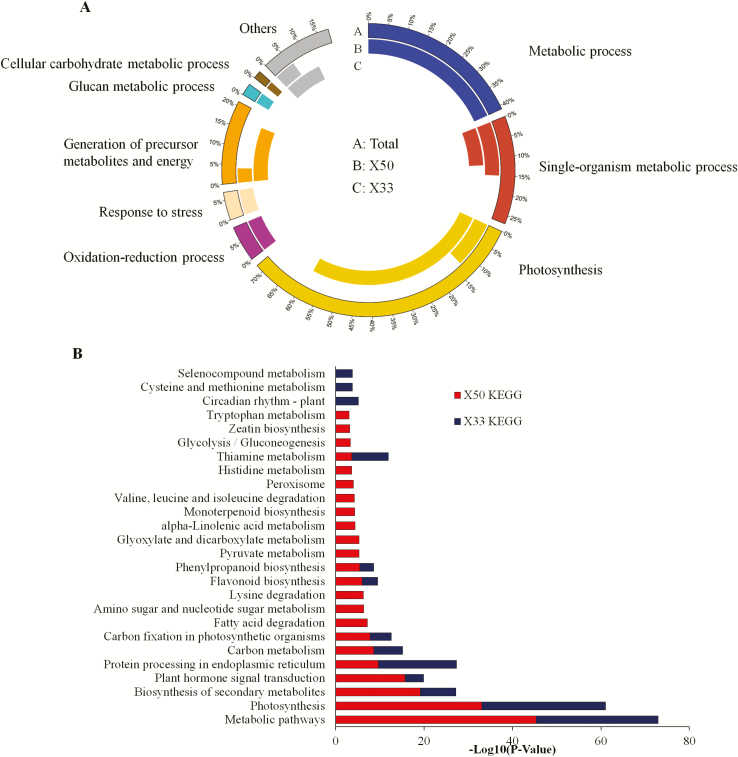
Analysis of GO (Gene Ontology) enrichments and KEGG (Kyoto Encyclopedia of Genes and Genomes) pathways for cotton cultivars X50 and X33 following treatment with either thidiazuron and ethephon (TE) or water (W, control). (A) GO enrichments (biological processes, FDR<0.001). (B) KEGG pathways (*P* <0.001). The data are for the functional classification of all DEGs at 1 d and 3 d following the defoliant treatment.

Gene Ontology (GO) classification was applied to the 2434 DEGs to identify the functional categories that were active in biological processes during defoliation. The biological processes of all the DEGs in the X50 and X33 cultivars were similar at the three time-points following TE treatment compared with the control (Supplementary Fig. S2). Considerable differences were found between X50 and X33 at 1 d and 3 d in the early days following TE treatment (Fig. 3A). In X50, most genes were associated with ‘metabolic process’, ‘oxidation-reduction process’, ‘photosynthesis’, and ‘response to stress’ following TE treatment. By contrast, only ‘photosynthesis’, ‘generation of precursor metabolites and energy’, and ‘metabolic process’ were associated with the TE treatment in X33.

KEGG pathway analysis was performed to explore the biological pathways of DEGs that were responding to the TE defoliant. At all three time-points, all the DEGs between the X50 and X33 cultivars expressed similar KEGG pathways (Supplementary Fig. S3). However, the DEGs between both the X50 and X33 cultivars expressed different KEGG pathways in the early days following TE treatment (1d and 3d). The DEGs were assigned to 23 KEGG pathways in X50 and only 13 KEGG pathways in cultivar X33 following TE treatment at 1 d and 3 d (Fig. 3B). Some pathways were the same, such as ‘metabolic pathways’, ‘photosynthesis’, ‘plant hormone signal transduction’, and ‘flavonoid biosynthesis’. Pathways unique in X50 included ‘phenylpropanoid biosynthesis’, ‘fatty acid degradation and metabolism’, ‘glycolysis/gluconeogenesis’, ‘tryptophan metabolism’, ‘peroxisome’, and ‘zeatin biosynthesis’. Based on the KEGG pathway and GO function analyses, we concluded that hormone metabolism, hormone signal transduction, genes for hydrolase and oxidoreductase activity, and some transcription factor genes were important for the plant response to the defoliant. A large number of the genes undergoing differential expression responses to the treatment belonged to Types I and III.

### The cytokinin signaling pathway participates in defoliation

**Fig. 4.**
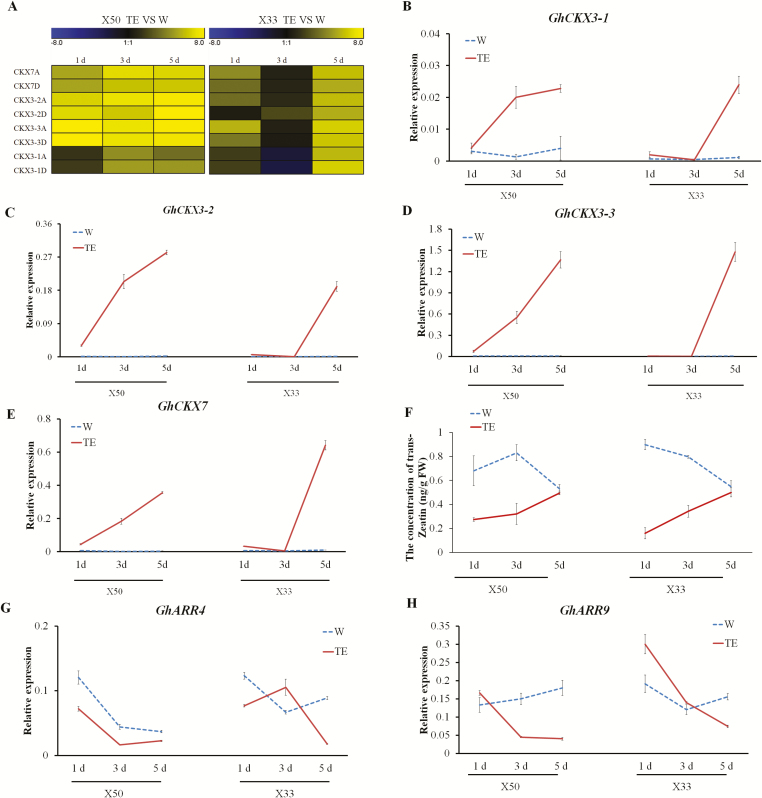
qRT-PCR validation of the expression of *GhCKX*s and the content of trans-Zeatin in the abscission zones of cotton cultivars X50 and X33 following treatment with either thidiazuron and ethephon (TE) or water (W, control) at different time-points. (A) *GhCKX* genes corresponding to the expression patterns in the RNA-seq data. (B–E) Relative expression levels of (B) *GhCKX3-1*, (C) *GhCKX3-2*, (D) *GhCKX3-3*, and (E) *GhCKX7* in X50 and X33 cultivars at 1 d, 3 d, and 5 d following treatment. (F) The content of trans-Zeatin. (G, H) Relative expression levels of (G) *GhARR4* and (H) *GhARR9*. The data are means (±SE), *n*=3.

The KEGG pathway analysis suggested that zeatin biosynthesis genes, including several cytokinin oxidase/dehydrogenase (*GhCKX*) genes, may play important roles in cotton defoliation following TE treatment. CKX is the enzyme that catalyses the catabolism of cytokinins to inactive products (Jones and Schreiber, 1997) and expression of *CKX* reduces the endogenous cytokinin content in plants (Werner *et al*., 2001, 2003). Seven *GhCKX* genes, including *GhCKX3* and *GhCKX7*, were differentially expressed during TE treatment in both the X50 and X33 cultivars, but with different expression patterns (Fig. 4A, Supplementary Table S4). qRT-PCR revealed that the expression of all the *GhCKX* genes was increased at 1 d after TE treatment in X50 but only after ~5 d in cultivar X33. Levels of endogenous cytokinins were decreased following TE treatment (Fig. 4F, Supplementary Fig. S4A–C). The cytokinin-response regulator (*ARR*) genes, which are induced by cytokinin, were found to be down-regulated by the TE treatment (Fig. 4G, H), leading us to speculate that cytokinin degradation pathways were activated following the treatment, potentially leading to a reduction in endogenous cytokinin levels.

**Fig. 5.**
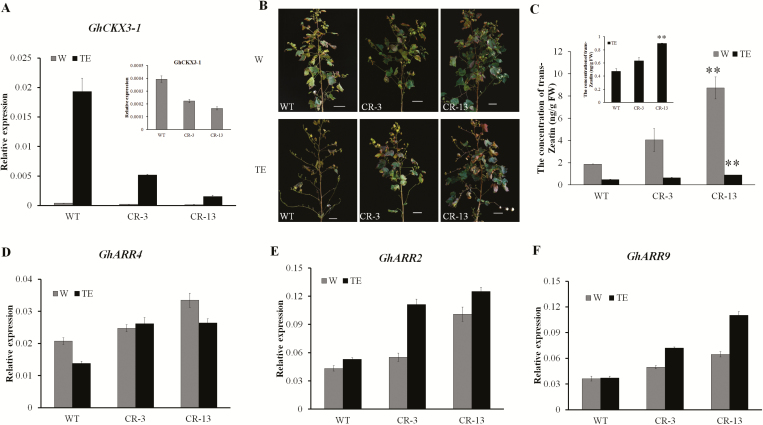
Suppression of *GhCKX3* in cotton results in delayed defoliation in response to treatment with either thidiazuron and ethephon (TE) or water (W, control). (A) qRT-PCR analysis of the relative expression of *GhCKX3* in *GhCKX3*-RNAi (CR-3, CR-13) and wild-type (WT) lines grown in the field at 3 d after treatment. The inset shows the results for the control (W) only for better clarity. (B) Representative images of plants at 10 d after treatment. (C) The content of trans-Zeatin in *GhCKX3*-RNAi lines and the WT at 3 d after treatment. The inset shows the results for TE only for better clarity. Significant differences compared with the WT were determined using Student’s *t*-test: **P<0.01. (D–F) qRT-PCR analysis of the relative expression of (D) *GhARR2*, (E) *GhARR4*, and (F) *GhARR9* at 3d following treatment. The data are means (±SE), *n*=3.

Two transgenic RNAi lines of *GhCKX3* (CR-3, CR-13) and their wild-type (WT) were obtained for functional analysis (Zhao *et al*., 2015) and were treated with TE at the boll-opening stage, with water treatment (W) as the control. The expression of *GhCKX3* was significantly increased at 3 d following TE treatment in the WT, but it was only slightly increased in the *GhCKX3*-RNAi lines (Fig. 5A). Most of the leaves were shed from the WT line at 10 d following TE treatment, while fewer leaves had been lost from the *GhCKX3*-RNAi lines (Fig. 5B). The concentration of cytokinins in the abscission zone was decreased in the WT compared with the *GhCKX3*-RNAi lines following TE treatment (Fig. 5C; Supplementary Fig. S4D). The *ARR* genes were up-regulated in the *GhCKX3*-RNAi lines compared with the WT (Fig. 5D–F). *GhARR2* and *GhARR9* had higher expression levels in the *GhCKX3*-RNAi lines following TE treatment than in the W control. The expression of *GhARR4* was decreased in the WT following TE treatment.

### Ethylene and its signaling pathway are involved in defoliation

**Fig. 6.**
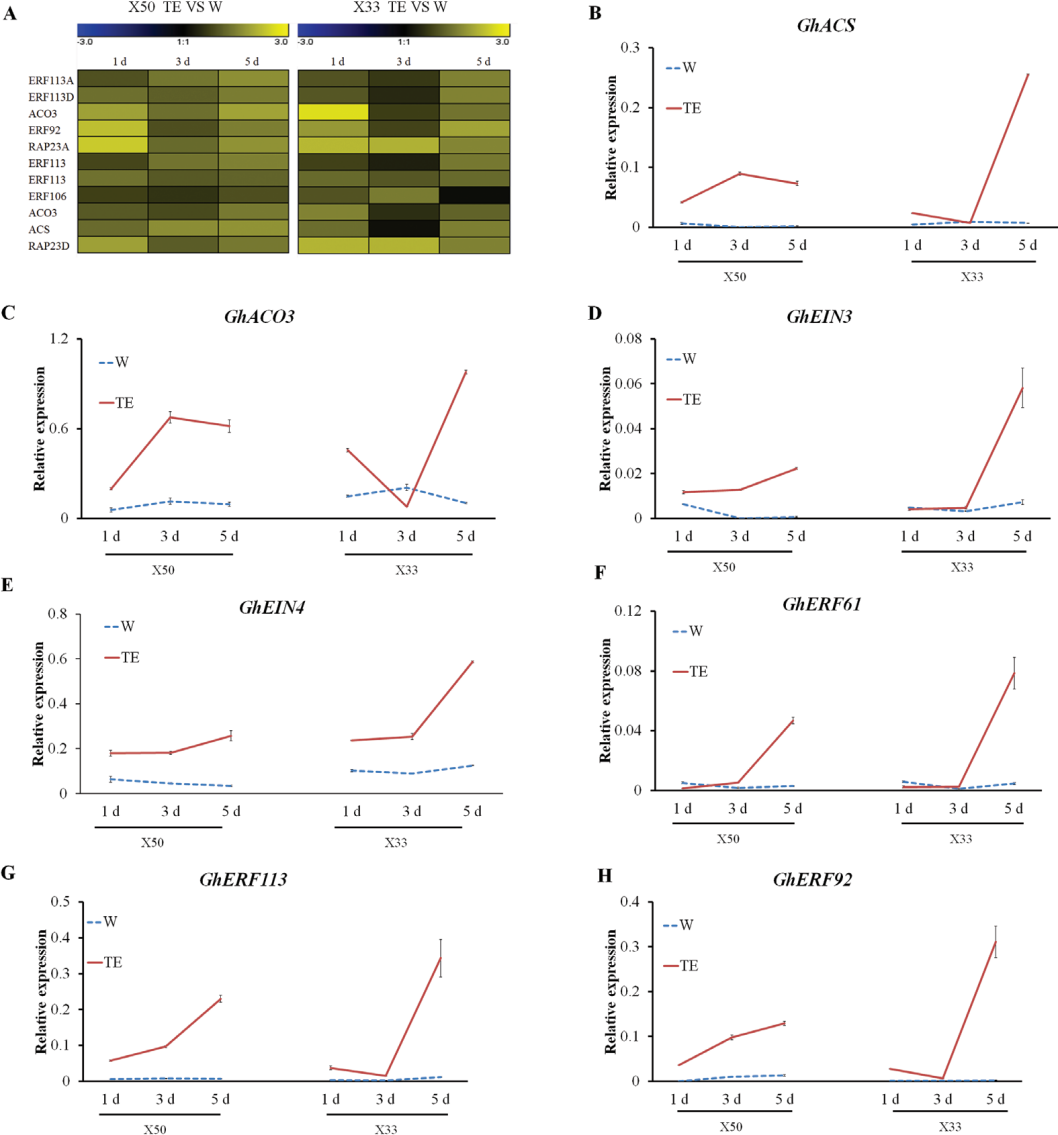
qRT-PCR validation of ethylene-related genes in the abscission zones of cotton cultivars X50 and X33 following treatment with either thidiazuron and ethephon (TE) or water (W, control) at different time-points. (A) Ethylene-related genes corresponding to the expression patterns in the RNA-seq data. (B–H) Relative expression levels of (B) *GhACS*, (C) *GhACO3*, (D) *GhEIN3*, (E) *GhEIN4*, (F) *GhERF61*, (G) *GhERF113*, and (H) *GhERF92* at 1, 3, and 5 d after treatment. The data are means (±SE), *n*=3.

Ethylene synthesis genes, signal transduction genes, and ethylene response factors were all differentially expressed following the TE treatment in both X50 and X33, and the expression patterns were similar between the two cultivars across the time-points (Fig. 6A, Supplementary Table S5). The qRT-PCR results confirmed that *ACC OXIDASE* (*GhACO3*) and *GhACS* were generally up-regulated in the two cultivars compared with the W control (Fig. 6B, C), together with the ethylene receptor gene *GhEIN3* (Fig. 6D) and some ethylene response genes (Fig. 6E–H). In X50, most of the genes showed consistent and increasing up-regulation across all the time-points, whereas the response was delayed in X33 with up-regulation only being observed at 5 d. Overall, the results indicated that enhanced ethylene signaling is a response to TE-induced defoliation.

**Fig. 7.**
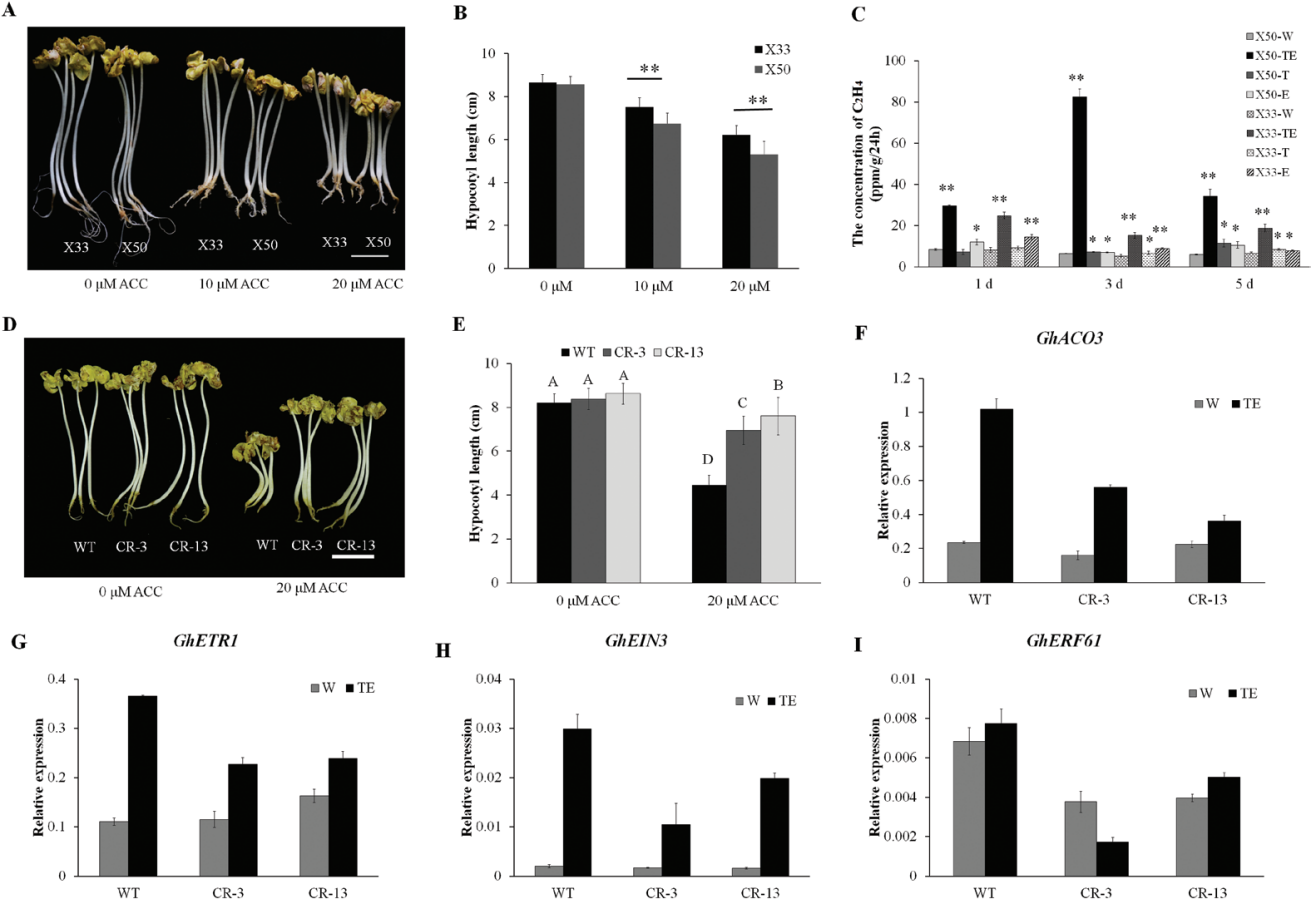
Ethylene-associated phenotypes and expression of ethylene signaling and biosynthesis genes in cotton cultivars X50 and X33 in response to treatment with either thidiazuron and ethephon (TE) or water (W, control). (A) Germinated seedlings were grown in the dark on Murashige and Skoog medium treated with either 0, 10, or 20 μM ACC for 5 d. Scale bars are 3 cm. (B) Hypocotyl elongation of the seedlings of X50 and X33 following ACC treatments. (C) Ethylene evolution in abscission zone explants at 1, 3, and 5 d following different defoliant treatments. T, thidiazuron alone; E, ethephon alone. Significant differences compared to the control (X50-W, X33-W) for each cultivar and time-point were determined using Student’s *t*-test: *P<0.05, **P<0.01. (D) Germinated seedlings of *GhCKX3*-RNAi lines and the wild-type (WT) were grown in the dark on Murashige and Skoog medium treated with either 0 μM ACC or 20 μM ACC for 5 d. Scale bars are 3 cm. (E) Hypocotyl elongation of the seedlings of *GhCKX3*-RNAi lines and WT following ACC treatments. Different letters indicate significant differences as determined by multiple comparisons using SPSS 17.0 (*P*<0.05). (F–I) Relative expression levels of the ethylene biosynthesis gene *GhACO3* (F) and ethylene signaling genes (G–I) in *GhCKX3*-RNAi lines and WT at 3 d after treatment. The data are means (±SE), *n*=3.

Based on the different responses to the TE defoliant, we then determined the sensitivity to ethylene in the two cultivars. Seedlings were treated with different concentrations of the ethylene precursor ACC. Ethylene induces the triple response in seedlings, namely reduced hypocotyl elongation, hypocotyl swelling, and exaggeration of the apical hook (Guzmán and Ecker, 1990), and this response was obvious in the dark-grown seedlings of X50 and X33 (Fig. 7A). In terms of reduction of hypocotyl length, the response was significantly enhanced in X50 compared with X33 (Fig. 7B). We therefore used gas chromatography to examine the production of ethylene in the two cultivars in the four different treatments (W, TE, T, E) across the different time points. TE treatment resulted in significantly more ethylene being produced in both cultivars relative to the control (W) (Fig. 7C), and the level in X50 was significantly higher than X33 at 3 d after treatment (*P*<0.05). The results suggested that cultivar X50 was both more sensitive to ethylene and produced more of it following TE treatment. The amounts of ethylene produced in response to T and E alone were higher than in the control, but were much less than in the combined TE treatment, suggesting a synergistic effect of the components on ethylene biosynthesis.

To examine interactions between the ethylene and cytokinin signaling pathways in the response to TE treatment, seedlings of *GhCKX3*-RNAi transgenic lines and their WT were treated with ACC. After 5 d of growth in the dark with 20 μm ACC, the WT had thicker and shorter hypocotyls compared with the *GhCKX3*-RNAi plants, i.e. the transgenic lines had weaker triple-response phenotypes (Fig. 7D, E). When measured at 3 d following TE treatment, expression of the ethylene biosynthesis and signaling genes *GhACO3*, *GhETR1*, *GhEIN3*, and *GhERF61* (Fig. 7F–I) was higher in the WT than the *GhCKX3*-RNAi lines. These results suggested that down-regulation of the expression of *GhCKX3* reduced the response to ethylene and the TE defoliant in cotton.

### Defoliation is accompanied by transcription regulation, hydrolysis, and redox processes

A number of transcription factor (TF) genes were found to be differentially expressed in X50 and X33 following the TE treatment. For example, the WRKY, MYB, and NAC TF genes were up-regulated following defoliant treatment compared with the control, with Type-III expression patterns (Supplementary Fig. S5, Supplementary Table S6). The expression of most of these TF genes was significantly increased in X50 during the early period following treatment, whereas in X33 the increase was delayed. MYB TF genes were down-regulated at 3 d in X33. In contrast, some TF genes clustering to Type I, including auxin-response factors, homeobox-leucine zipper proteins, zinc finger proteins, and protein LHY, were down-regulated following TE treatment compared with the control in both cultivars. Again, a delay in the response could be seen in X33 compared with X50.

Hydrolase activity genes may play roles in cell wall and carbohydrate metabolism. Oxidoreductase activity genes participate in oxidation–reduction processes and responses to stress. We identified genes encoding hydrolase activity acting on glycosyl residues, including glucan endo-1,3-beta-glucosidase, endochitinase 1, endoglucanase 1, and Beta-D-xylosidase 1, which are important for cell wall structure, and which also clustered to the Type-III pattern (Supplementary Fig. S6A–D). The hydrolase genes were up-regulated following TE treatment at 3 d in X50.

Peroxidases were also differentially expressed as a result of TE treatment, including *PEROXIDASE 4*, *PEROXIDASE 16*, *PEROXIDASE 21*, *PEROXIDASE 45*, *PEROXIDASE 53*, *PEROXIDASE 55*, and *PEROXIDASE 61*, and clustered to the Type-III pattern (Supplementary Fig. S5). These genes are responsive to oxidative stress and participate in oxidation–reduction processes, and were also up-regulated (Supplementary Fig. S6E, F), This change in expression was accompanied by an increase in endogenous H_2_O_2_ content at 5 d following TE treatment compared with the control (Supplementary Fig. S7).

## Discussion

### Thidiazuron and ethephon accelerate the activation of the leaf abscission zone

Chemical defoliation enhances the efficiency of mechanical harvesting of cotton. Therefore, research on the process of leaf abscission, namely the response and activation of abscission at the biochemical and molecular levels, is of high importance. Thidiazuron, a synthetic cytokinin analog, has been shown to reduce basipetal auxin transport in petiole segments and to enhance endogenous ethylene evolution during leaf abscission (Suttle, 1985, 1988). Coronatine (COR), a bacterial toxin containing an analog of methyl jasmonic acid, is a potential defoliant that modulates the expression of genes involved in ethylene signaling and cell wall hydrolases to induce cotton leaf abscission (Du *et al*., 2014). Auxin and ethylene are considered to contribute to cell separation, but only high concentrations of ethylene applied to plants inside desiccators can induce rapid defoliation (Mishra *et al*., 2008*b*), which is clearly not conducive to machine-picking in the field.

Spraying a mixture of thidiazuron and ethephon (TE) on the cultivar X50 causes green leaves to be shed rapidly and improves the efficiency of machine-picking. Our results showed that cotton leaf abscission was most accelerated by treatment with TE, followed by a moderately efficient defoliation rate following treatment with thidiazuron alone (T). In contrast, low defoliation rates were observed after treatment of X50 with ethephon alone (E). Histological sections revealed TE-induced abscission zones in treated leaves (Fig. 1), which were observed at 3–5 d following treatment. This phenotype was in accordance with defoliation rates observed in the field (Supplementary Table. S1). Thidiazuron is an effective defoliant, mediated in part by an increase in endogenous ethylene evolution (Suttle, 1985). Our results showed that thidiazuron and ethephon treatments alone each caused an increase in ethylene evolution from abscission zone explants; however, significantly more ethylene was produced following spraying with thidiazuron and ethephon together, and this was associated with a higher rate of defoliation in intact plants (Fig. 7C, Supplementary Table S1). The results showed that thidiazuron and ethylene have a synergistic effect on defoliation in cotton.

### Complex crosstalk between cytokinin and ethylene signaling in leaf abscission

RNA-seq showed that 1710 genes were clearly regulated by the TE treatment in the X50 cultivar, with 607 genes differentially expressed following treatment at 1 d and 863 genes at 3 d (Fig. 2A). Among the genes that were induced following TE treatment *CKX*s (Fig. 4), which encode enzymes that degrade cytokinins. The expression of *CKX*s is known to reduce the endogenous cytokinin content of plants and to result in diminished activity of the floral shoot apical meristems and reduced leaf growth. Cytokinins are known to play a central role in regulating development and cell division (Werner *et al*., 2001, 2003; Ashikari *et al*., 2005). The *ARR* genes mediate the transcriptional response to cytokinin, and their overexpression leads to a variety of phenotypes associated with cytokinin (Argyros *et al*., 2008; Ren *et al*., 2009).

Our data showed that TE treatment significantly induced the expression of *CKX3* and *CKX7* and reduced levels of cytokinin compared with the control in X50 (Fig. 4). In addition, some type-A *ARR* genes were down-regulated. Ethylene has a positive effect on the expression of *CKX* genes and hence increase the degradation of cytokinin. Suppression of *GhCKX3* by RNAi increased the expression of *ARR* genes, especially *GhARR4*, as well as reducing defoliation following TE treatment (Fig. 5). Suppression of *GhCKX3* is known to result in an enhancement of endogenous cytokinins (Zhao *et al*., 2015). The levels of cytokinin in WT plants were significantly reduced compared the *GhCKX3*-RNAi lines following TE treatment, and the expression pattern of *GhARR4* was consistent with the change of cytokinin (Fig. 5). *GhARR4* might therefore be an important factor in the chemical-mediated abscission through cytokinin signaling. In addition, suppression of *GhCKX3* decreased the expression of ethylene-related genes following TE treatment. In contrast, in the WT the expression of ethylene-related genes, including the synthesis gene *GhACO3* and some signal transduction genes, was increased following TE treatment compared with the control (Fig. 7). We therefore hypothesized that suppression of *GhCKX3* inhibited ethylene synthesis and signal transduction, thus reducing the sensitivity to ethylene.

In agreement with this, some genes related to ethylene synthesis, ethylene signal transduction, and ethylene response were up-regulated in X50 following the TE treatment compared with the control (Fig. 7). TE treatment led to the highest expression levels of ethylene-related genes and the highest endogenous ethylene evolution. Thidiazuron alone could promote ethylene synthesis and response in X50. RNA-seq of X33, which is relatively insensitive to defoliants, showed that *CKX3* and *CKX7* and ethylene-related genes had similar expression patterns to X50, with the expression of these genes increasing more slowly and the abscission zone appearing later in X33. Our results show that crosstalk between cytokinin and ethylene signaling play an important role in cotton defoliation.

### Transcription factors and leaf abscission

In addition to known TE treatment-induced gene expression, there were many groups of genes that were over-represented in our data set, of which TFs were particularly interesting (Supplementary Fig. S5). Some WRKY TF genes, especially *WRKY6* and *WRKY75*, play positive roles in leaf senescence. *WRKY75* is known to promote leaf senescence and to enhance H_2_O_2_ accumulation (Robatzek and Somssich, 2001; Li *et al*., 2012; Guo *et al*., 2017). It was also significantly induced by TE treatment and might therefore participate in the ethylene signaling and oxidation–reduction processes to regulate cotton defoliation. R2R3 MYB TF genes play an important role in regulating separation of the abscission zone in cassava (Liao *et al*., 2016*b*) and Arabidopsis *R2R3-AtMYB102* is rapidly induced in response to osmotic stress (Denekamp and Smeekens, 2003). In apple, *MdNAC047* is significantly induced by salt stress and regulates the expression of ethylene-responsive genes (An *et al*., 2018). Most of the *MYB* and *NAC* TF genes that we detected were up-regulated by TE treatment (Supplementary Fig. S5) and they might be involved in responses to stress to regulate cotton defoliation. In contrast, zinc finger proteins are negatively regulated during the formation of the abscission zone; For example, overexpression Arabidopsis *AtZFP2* delays abscission (Cai and Lashbrook, 2008). *ZFP8* and *ZFP6* are cytokinin-inducible genes that regulate cell differentiation programs during different developmental stages (Gan *et al*., 2007; Zhou *et al*., 2013). The increase in activity of cellulases and decrease in break strength in the abscission zone are strongly suppressed by auxin, and exogenous application negatively regulates and delays leaf abscission (Mishra *et al*., 2008*a*; Jin *et al*., 2015). We found that some auxin response factors were down-regulated by TE treatment (Supplementary Fig. S5.

### The cell wall in leaf abscission

Among the differentially expressed genes that we identified, several were found to be associated with defoliation, such as hydrolase genes, including polygalacturonase, endoglucanase, β-glucosidase, β-galactosidase, and β-D-xylosidase, which function to modify of the cell wall. Polygalacturonase is thought to be important for pectin degradation during cell separation, and is expressed in the abscission zone (Rhee and Somerville, 1998; Ogawa *et al*., 2009). Xylosidase is involved in cell wall organization (Kim *et al*., 2018) and β-glucosidase also mediates hydrolysis of Glc-conjugated ABA (ABA-GE) to increase the level of ABA in Arabidopsis (Xu *et al*., 2012). Some oxidative stress-related peroxidase genes were preferentially expressed following TE treatment (Supplementary Fig. S6). Peroxidase plays an important role in lignin formation. PEROXIDASE4 is involved in cell wall lignification and *peroxidase52* knock-out mutants have reduced lignin (Barros *et al*., 2015; Fernández-Perez *et al*., 2015). The catalysis and oxidation of peroxidases are responsible for lignin formation, and regulation of the structure of lignin promotes precise cell separation (Lee *et al*., 2018).

In summary, ethylene and cytokinin are regulators of leaf abscission in cotton. Thidiazuron and ethephon increase the endogenous production of ethylene and the degradation of endogenous cytokinin and result in cell wall degradation and precise cell separation. This is accompanied by regulation by transcription factors and changes in levels of reactive oxygen species.

## Supplementary data

Supplementary data are available at *JXB* online.

Table S1. Defoliation rates for X50 and X33 at 7 d following treatments in the field.

Table S2. Summary of RNA-seq and comparison with the *Gossypium hirsutum* reference genome.

Table S3. Normalized expression levels of DEGs between X50 and X33 following TE treatment at different time-points.

Table S4. The expression patterns of *GhCKX* genes in the RNA-seq data.

Table S5. The expression patterns of ethylene-related genes in the RNA-seq data.

Table S6. The expression patterns of DEGs for transcription factors and cell wall-related genes in the RNA-seq data.

Table S7. List of primers used in this study.

Fig. S1. Cluster analysis of 2434 DEGs between X50 and X33 at three time-points based on the K-means method.

Fig. S2. Functional classification of all DEGs according to GO enrichments.

Fig. S3. Functional classification of all DEGs according to KEGG pathways.

Fig. S4. The relative content of cytokinins in abscission zones in cultivars X50 and X33 following TE treatment in the field.

Fig. S5. DEGs corresponding to the expression patterns in the RNA-seq data.

Fig. S6. qRT-PCR analysis of DEGs in X50 at different time-points following TE treatment.

Fig. S7. H_2_O_2_ content in X50 at different time-points following different defoliant treatments.

Supplementary Figures S1-S7

Supplementary Tables S1-S7
