# Supplementary material for: Crosstalk between cytokinin and ethylene signaling pathways regulates leaf abscission in cotton in response to chemical defoliants
Source: J Exp Bot. 2019 Feb 4;70(5):1525–38. doi: 10.1093/jxb/erz036 (PMC6411381; doi:10.1093/jxb/erz036)

Fig. S1. Cluster analysis of 2,434 DEGs between X50 and X33 at three time points based on the K-means method. The DEGs were divided into three types.

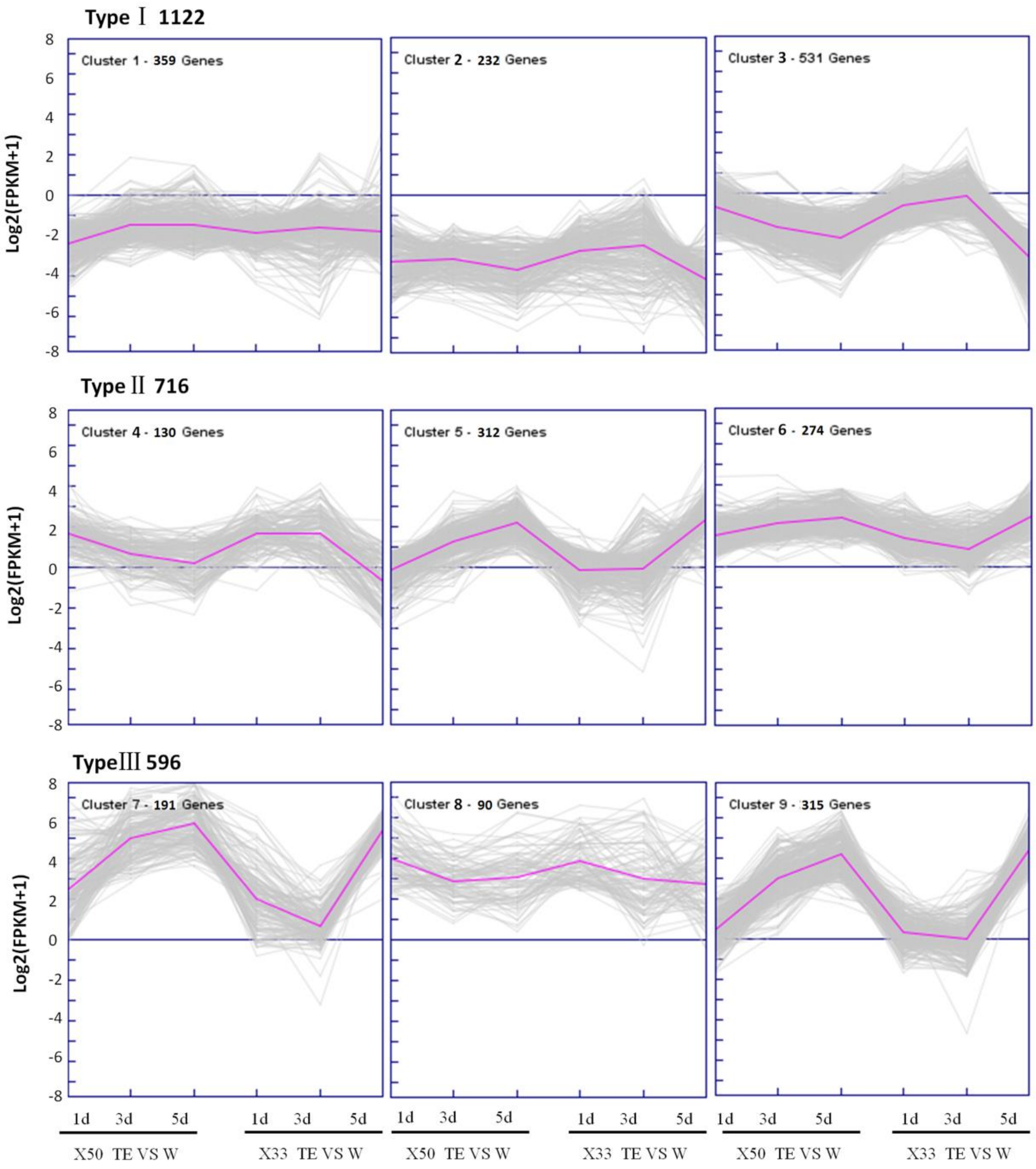

Fig. S2. Functional classification of all DEGs by GO enrichments. GO enrichments (biological processes, FDR < 0.001) with all DEGs following TE treatment for 1 d, 3 d and 5 d compared with W treatment in total of X50 and X33 (A) and respectively in X50 (B) and X33 (C).

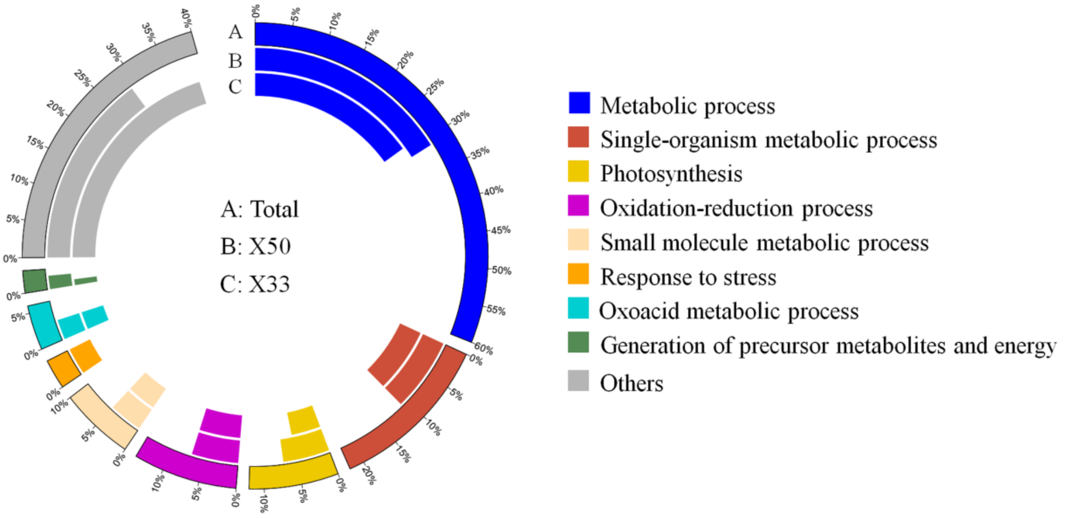

Fig. S3. Functional classification of all DEGs by KEGG pathway. KEGG pathway (P-Value < 0.001) with all DEGs following TE treatment for 1 d, 3 d and 5 d compared with W treatment in X50.

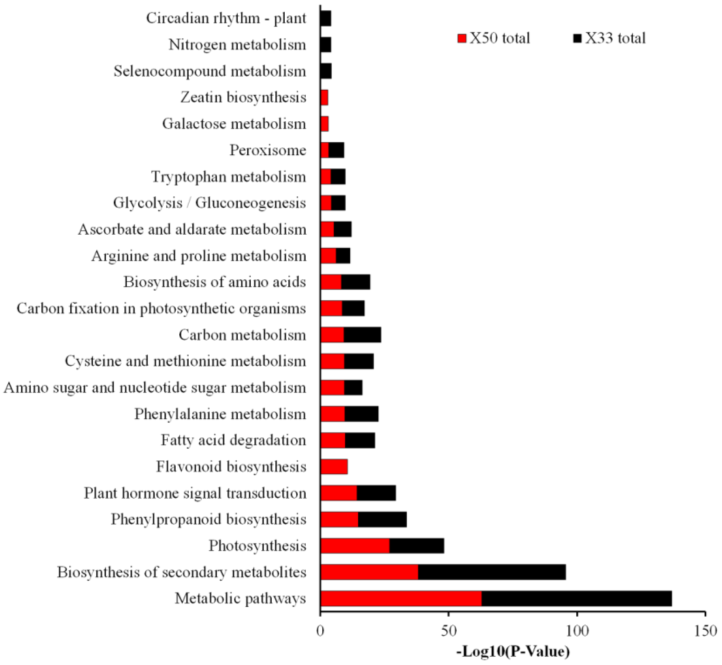

Fig. S4. The relative content of cytokinins in abscisson zones. The relative content of IPR (A) , ZR (B), IP (C) in X50 and X33 following TE and W treatments in the field relative to X50 following TE treatment for 1d. (D) The relative content of cytokinins (IPR, IP, ZR relative to WT treated with TE) in *GhCKX3* suppressed transgenic (CR-3, CR-13) and WT line following TE and W treatments for 3 d in the field. IPR: isopentenyl, ZR: trans-zeatin riboside, IP: isopentenyl adenine.

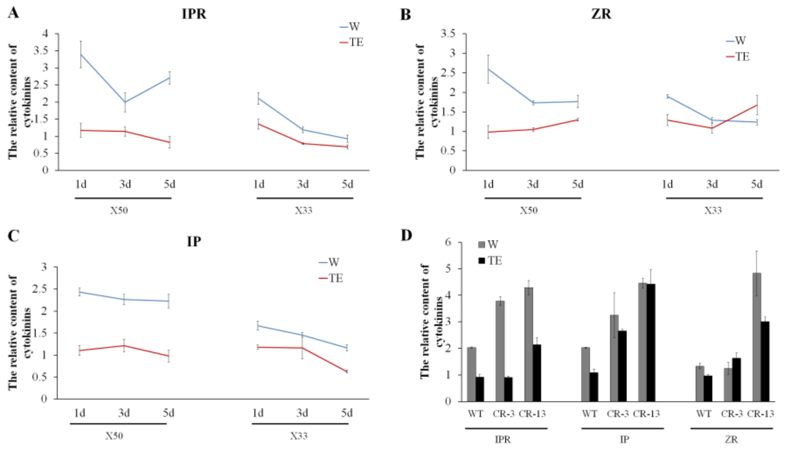

Fig. S5. DEGs corresponding to the expression patterns in the RNA-seq data. The DEGs included WRKY, MYB, NAC, LHY transcription factors, peroxidase, hydrolase, auxin-associated genes, homeobox-leucine zipper proteins, zine finger proteins.

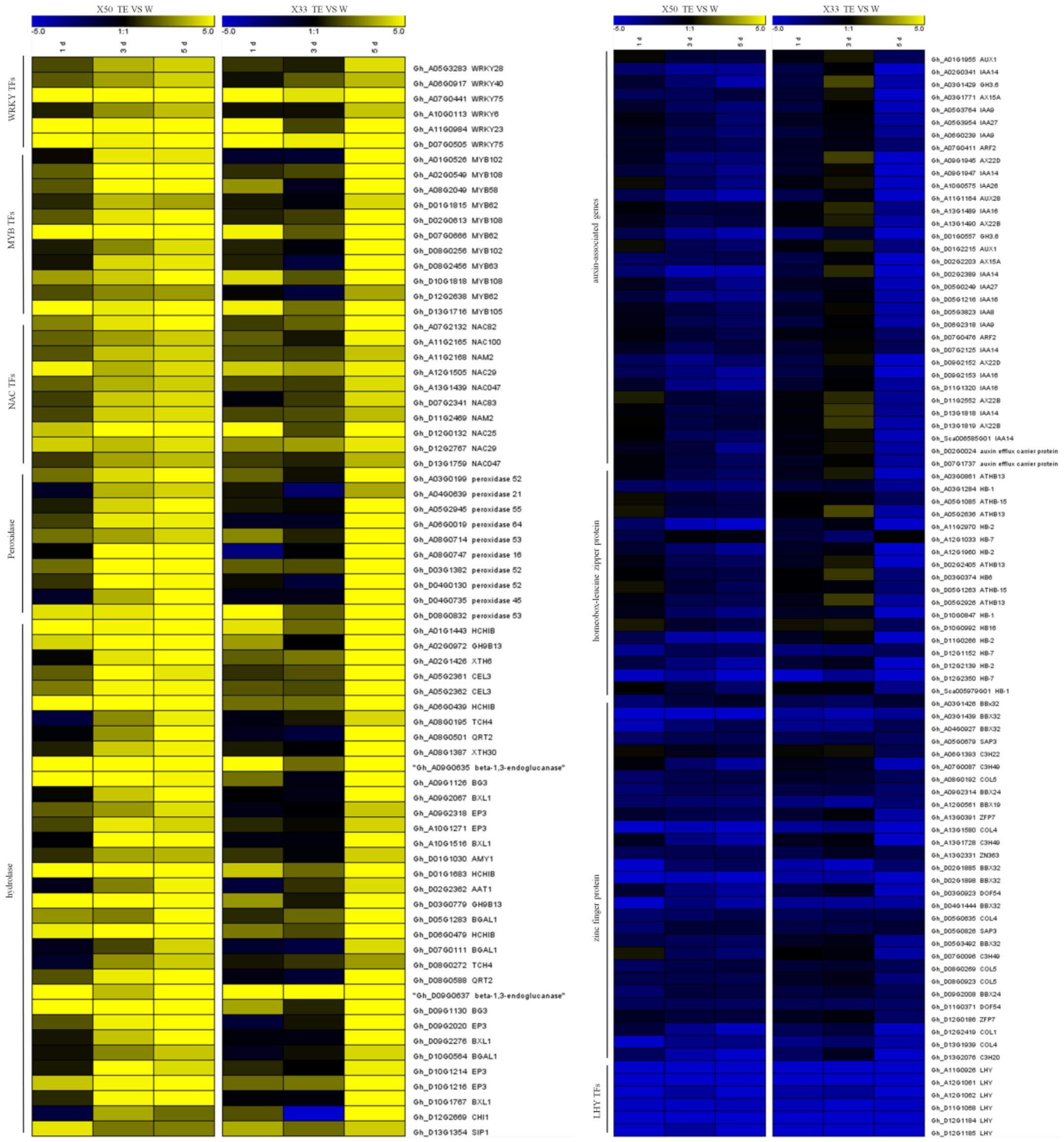

Fig. S6. qRT-PCR analysis of DEGs expression in X50 following different defoliant (W, TE) treatments at 1 d, 3 d and 5 d. And the expression of the genes were virtually absent in the control treatment (W) and up-regulated significantly following TE treatment. (A-D) The expression of DEGs related to cell wall structure. (E-F) The expression of peroxidase genes.

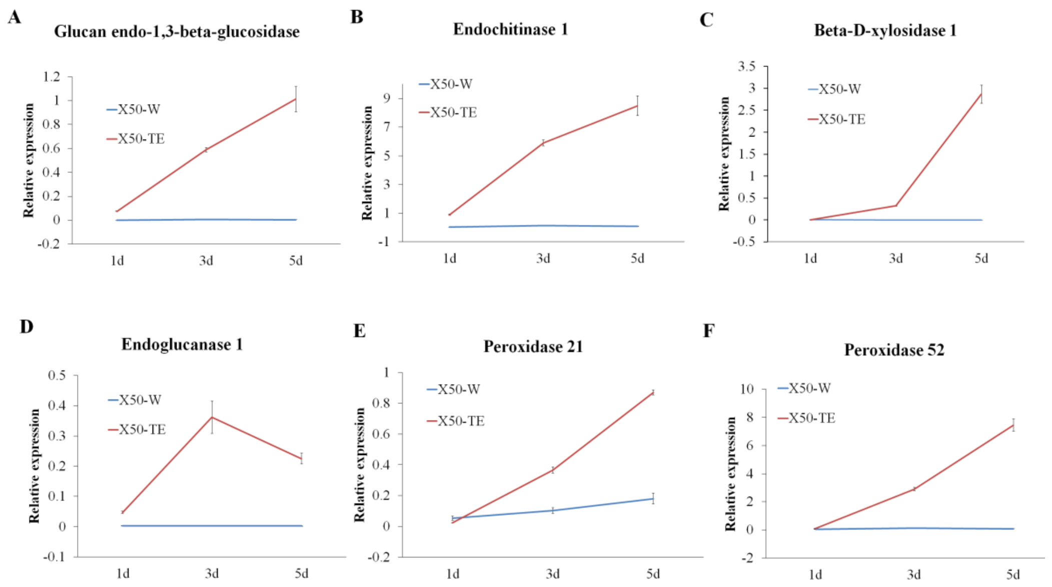

Fig. S7. The H_2O_2 content in X50 following different defoliant (W, TE, T ,E) treatments at 1 d, 3 d and 5 d.

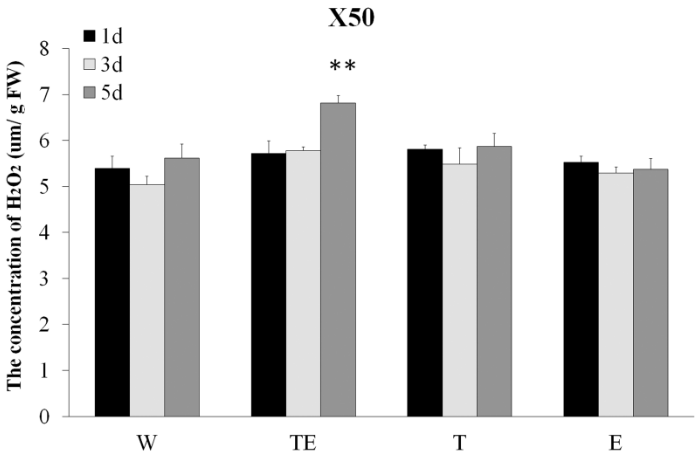

Supplement: Supplementary Figures S1-S7 [file erz036_suppl_supplementary_figures_s1-s7.pdf]
